# RdRp-based sensitive taxonomic classification of RNA viruses for metagenomic data

**DOI:** 10.1093/bib/bbac011

**Published:** 2022-02-07

**Authors:** Xubo Tang, Jiayu Shang, Yanni Sun

**Affiliations:** Department of Electrical Engineering, City University of Hong Kong, Tat Chee Avenue, Kowloon, Hong Kong, China SAR; Department of Electrical Engineering, City University of Hong Kong, Tat Chee Avenue, Kowloon, Hong Kong, China SAR; Department of Electrical Engineering, City University of Hong Kong, Tat Chee Avenue, Kowloon, Hong Kong, China SAR

**Keywords:** RNA virus, RNA-dependent RNA polymerase, Probabilistic Relational Neighbor Classifier, Graph Neural Network

## Abstract

With advances in library construction protocols and next-generation sequencing technologies, viral metagenomic sequencing has become the major source for novel virus discovery. Conducting taxonomic classification for metagenomic data is an important means to characterize the viral composition in the underlying samples. However, RNA viruses are abundant and highly diverse, jeopardizing the sensitivity of comparison-based classification methods. To improve the sensitivity of read-level taxonomic classification, we developed an RNA-dependent RNA polymerase (RdRp) gene-based read classification tool RdRpBin. It combines alignment-based strategy with machine learning models in order to fully exploit the sequence properties of RdRp. We tested our method and compared its performance with the state-of-the-art tools on the simulated and real sequencing data. RdRpBin competes favorably with all. In particular, when the query RNA viruses share low sequence similarity with the known viruses ($\sim 0.4$), our tool can still maintain a higher F-score than the state-of-the-art tools. The experimental results on real data also showed that RdRpBin can classify more RNA viral reads with a relatively low false-positive rate. Thus, RdRpBin can be utilized to classify novel and diverged RNA viruses.

## Introduction

Eukaryotic viruses are abundant and diverse. Based on the recent report of IPBES Workshop on Biodiversity and Pandemics [33], it is estimated that $\sim $1.7 million viruses inhabit mammal and avian hosts, and 631 000−827 000 of them may infect humans. Although many viruses are commensal, some viruses, especially RNA viruses, have caused notorious infectious diseases, such as SARS, Ebola, COVID-19, etc. Many RNA viral pathogens are zoonotic viruses that have animals as their native hosts. It is estimated that about 75% of new human diseases are caused by microbes originating in animals [12]. To prepare for future breakouts of new infectious diseases, it is important to conduct viral composition analysis from a wide range of environmental niches and host-associated samples.

Metagenomic sequencing, which can sequence all the microorganisms present in one sample, has become the favored approach for virus discovery [6]. In particular, it enables viral genome discovery without prior knowledge of the sequence for primers, allowing us to study both known and novel viruses.

However, it is not trivial to characterize RNA viruses from metagenomic data. There are two major challenges. First, the reference database is far from being complete, which can lead to a low recall of comparison-based methods. Second, RNA viruses in ecosystems, such as the marine water, are more diverse than currently characterized viruses [23]. Newly identified viruses or viral genes may not share any significant sequence similarity with the reference genomes, which can fail the most powerful sequence comparison methods. Thus, methods beyond sequence comparison are in great need to conduct sensitive taxonomic classification for new viruses.

Due to the complexity of metagenomic data, most pipelines for viral analysis combine reference-based classification and *de novo* assembly. Some conduct *de novo* assembly first and compare the contigs with references for phylogenetic analysis [22]. The others classify reads into different taxonomic groups using reference-based methods and then conduct assembly for each group [15, 16]. While these virus identification tools made significant contributions in purifying the data by removing non-virus reads and classifying virus-like reads into functional/taxonomic groups, their performance heavily depends on high-quality viral references. However, quality viral references are not always available for new and host-switching viruses. Thus, there is a need for new methods that can classify both closely and remotely related RNA viruses. In this work, we will introduce a new method that capitalizes on the utilities of alignment-based and learning-based methods for maximizing the sensitivity of read-level taxonomic classification. Before we detail our method, we first summarize the related work.

### Related work

Composition analysis can be conducted at read level or contig level. Although contigs contain more information than short reads, metagenomic assembly is still computationally challenging and can be error-prone for complex data. Thus, read binning, which clusters reads of the same origin, is also a popular method for composition analysis at read level [15, 16]. In this work, we will conduct composition analysis.

For metagenomic data taxonomic classification, there are two types of methods: reference-based and marker-based. Basic Local Alignment Search Tool (BLAST) [1] is a tool that has been commonly used in aligning the unknown biological sequences against the reference database. But it is too computationally expensive to classify a large number of metagenomic sequencing reads. To improve the efficiency, DIAMOND [4] builds indexes for the protein references and queries simultaneously, achieving up to 10 000x speedup compared with BLAST. To get the taxon of reads, Kaiju [18] uses the minimum exact match, and MMseqs2 [25] and Kraken2 [31] use k-mer matching. However, the performance of reference-based methods will decrease with the increase of the distance between the test set and the reference database, or when the reference database is incomplete.

The second type of method is marker-based and has shown better profiling performance in taxonomy classification [3, 26]. Because the marker-based reference database is much smaller, it leads to higher efficiency. MetaPhlAn3 [3] is a marker-based tool that contains 1.1M marker genes. It achieves better performance than other profiling tools with lower time and memory consumption. Core-Kaiju is a tool that combines Kaiju and the core protein family to improve precision. However, although these tools have achieved excellent performance on the composition analysis, most of the tests were conducted on bacteria. Because RNA viruses and bacteria have highly different sequence compositions and gene organization, different batches of efforts are needed for choosing and using the marker genes from RNA viruses.

### Overview of our method

Because previous works have shown that using marker-based genes can improve the classification performance, we will design our taxonomic classification model based on RNA-dependent RNA polymerase (RdRp) genes, which are responsible for replicating the genome and performing transcription [30] in RNA viruses. RdRp gene exists in almost all RNA viruses, except retroviruses, such as human immunodeficiency viruses. As the only universal gene among RNA viruses, researchers use RdRps to study the phylogeny relationship of RNA viruses [23, 30]. In this work, we focus on order-level taxonomic classification for RNA viruses. The order-level labels for available RNA viruses are relatively stable. According to ICTV, the number of taxa of RNA viruses changed from 28 to 29 at the order level, and 102 to 111 at the family level from 2019 to 2020 [28]. In addition, as we will use learning-based methods, order-level classification allows us to obtain more training data. When more RNA viruses are sequenced and classified, our framework can be conveniently adjusted to lower ranks.

In this study, we developed a novel method named RdRpBin, which combines the reference-based method and graph learning, to identify and classify RNA virus reads in metagenomic data. Unlike tools that only classify sequences with alignments against a reference database, we can classify RdRp reads that cannot be aligned to the database by two strategies. One is to use label propagation to get the labels by building edges between test reads, and the other is to build a graph through motifs and then classify reads by Graph Convolutional Network (GCN). We tested our model using simulated test datasets with different similarities against the reference database. Then we compared the performance with other tools on real data. The results showed that our method can find more RNA viral reads in the metagenomic data without jeopardizing the precision.

## Method

### Sequence properties of RdRp

**Figure 1:**
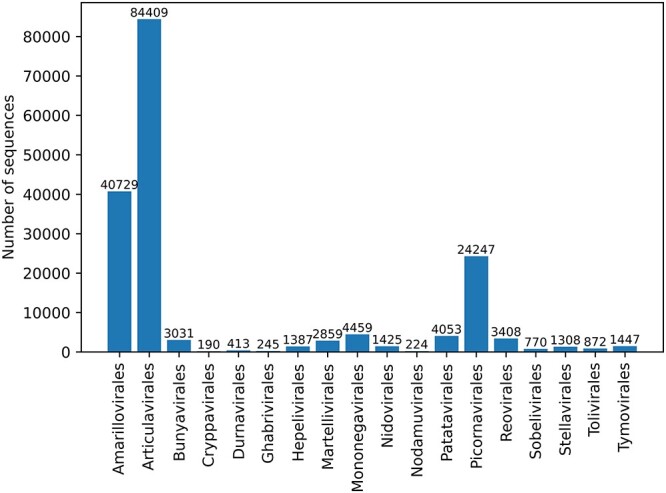
The number of RdRps in different orders.

We downloaded all the RdRp families from Pfam [19], which contains protein families encoded by profile hidden Markov models (pHMMs). In order to obtain as many RdRps as possible, we used all RdRps in NCBI that can be aligned with RdRp pHMMs, including the segmented RdRps such as those in H1N1. Then, we kept orders containing at least 150 RdRps to build the database called NCBI-RdRp, which contains 18 orders and 175 476 RdRps. The numbers of RdRps in different orders are shown in Figure 1.

**Figure 2:**
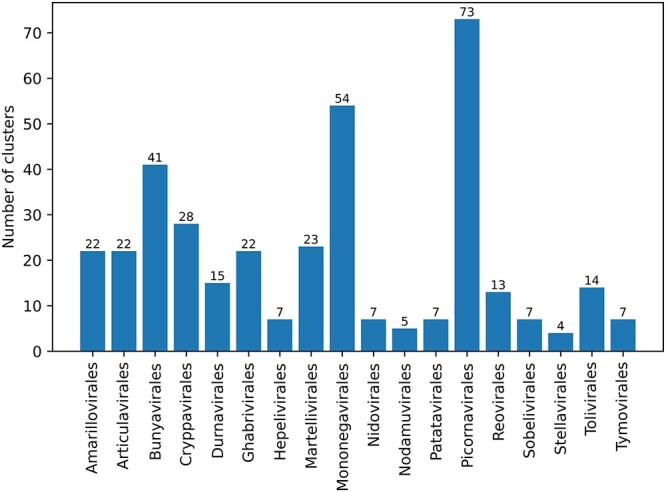
The number of clusters after using CD-HIT with the identity threshold of 0.4.

Although using marker genes has achieved better classification performance based on the empirical results of a number of tools, we must incorporate the property of RdRp sequences into the method design in order to maximize the sensitivity of read classification. First, RdRps of the same taxon do not always exhibit high similarity [27]. To quantify the similarity between RdRp within the same order, we use CD-HIT [14] to cluster RdRp sequences according to the sequence similarities in NCBI-RdRp. Figure 2 summarizes the number of clusters at an identity percentage of 0.4. The result shows that the three most diverse orders are *Picornavirales*, *Mononegvirales* and *Bunyavirales*.

Second, the number of RdRps in different orders and clusters vary greatly (as shown in Figures 1 and 2).

**Figure 3:**
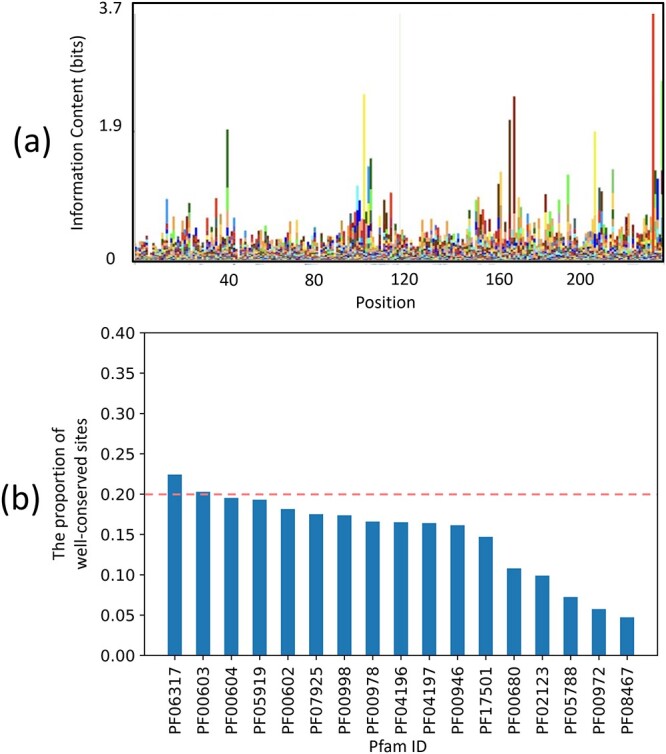
**(A)** The HMM logo of PF00680 from position 0 to 236, which is generated by Skylign [29]. **(B)** The proportion of highly conserved positions in different Pfam families.

Third, the conservation along RdRp is also heterogeneous. Figure 3  **(A)** shows part of the HMM logo for PF00680, an RdRp Pfam family. The height, which represents position-specific conservations, changes along with different sites. For reads that are sequenced from poorly conserved regions, alignment-based methods tend to miss them. To examine whether other RdRp genes also have heterogeneous conservation, we computed the percentage of well-conserved sites in the RdRp protein families. If a site with the three most frequent amino acids has a summed frequency above 0.6, it is defined as a well-conserved site. The result is summarized in Figure 3  **(B)**, which shows that almost all Pfam families’ highly conserved sites are below 20%.

### Overview of the algorithm

**Figure 4:**
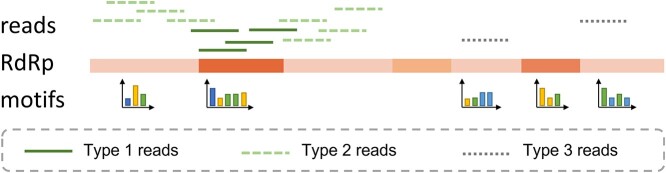
Three types of reads that can be classified by our method. The darker the color on RdRp, the higher the conservation.

The sequence properties of RdRp can lead to three types of reads as shown in Figure 4. The first type of reads are sequenced from well-conserved regions of RdRp and can be effectively classified using an aligned-based strategy. The second and third types of reads are both sequenced from the poorly conserved regions but reads from type 2 share overlaps or high similarities with reads in type 1.

**Figure 5:**
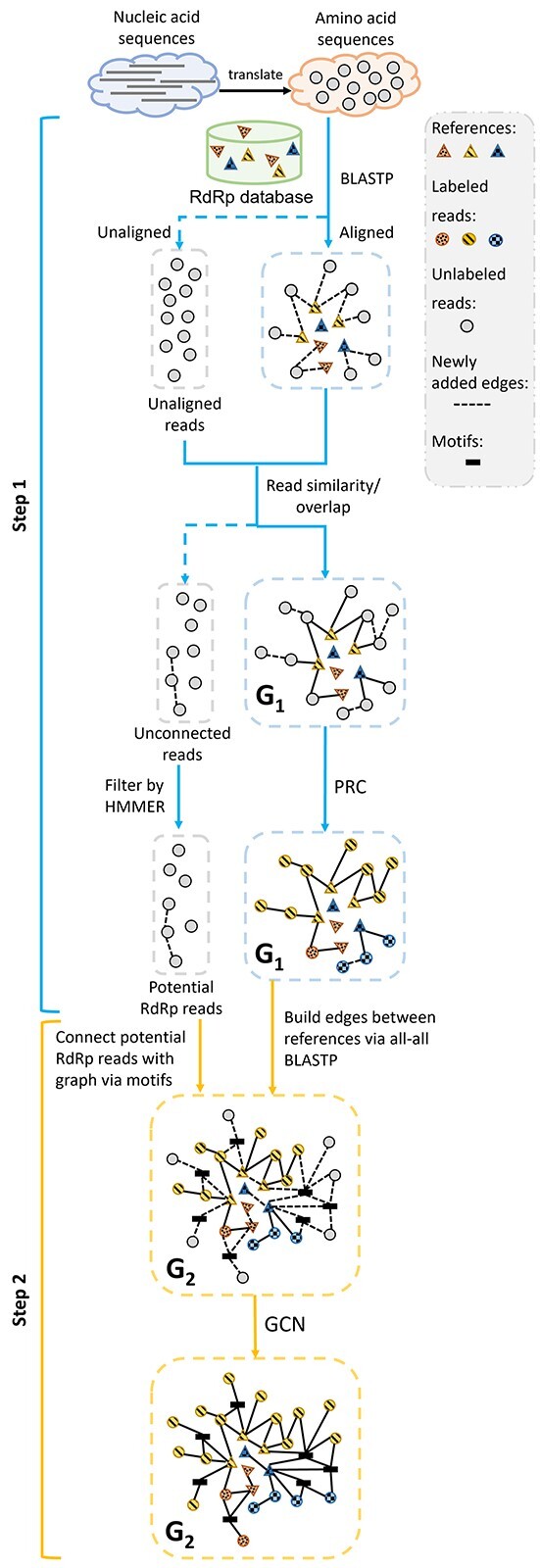
The pipeline of the RdRp identification and classification. In Step 1, we build $G_1$, which consists of two types of edges: the reads aligned to the references and the similarity/ overlaps between reads. We apply Probabilistic Relational Neighbor Classifier (PRC) to get the taxa of these reads. For the unconnected reads, we will use HMMER to detect potential RdRp reads. In Step 2, we augment $G_1$ by adding motifs from RdRp and edges for reads that can match the motifs. Then, we predict the taxa of the potential RdRp reads by GCN.

To classify all types of reads, our method consists of two steps. In the first step, we will build a graph containing queries and references based on sequence similarity, and use a relational classifier to classify the highly conserved queries and as many poorly conserved queries as possible. In the second step, we will focus on classifying reads that cannot be classified in the first step. Motivated by the model text GCN [32], we will create a motif GCN, which adds motifs as the nodes in the graph and motif-matching as the edges. Then, the graph convolution will be conducted for read node classification. To alleviate the bias caused by data imbalance, we assign larger weights to small classes when training models. The pipeline is sketched in Figure 5.

Our tool can take either DNA or protein sequences as input. For the DNA sequences, we will translate the sequences (i.e. reads) into proteins with six reading frames. Then, we only keep the translations without stop codons, which is equivalent to the longest translation for a read. The shortcoming of this method is that we will discard the reads that are sequenced from the end of RdRps. Thus, RdRpBin also allows the user to specify a length cutoff for the translated amino acid sequences if none of the translations are complete for a read. The translations with length above the cutoff will be kept for downstream analysis.

#### Step 1: Using Probabilistic Relational Neighbor Classifier to classify reads

In the first step, we build a sequence similarity graph $G_1$, where the nodes are the translated reads and the references. The edges are built in two steps. First, we will build edges between reads and reference RdRp database using DIAMOND BLASTP (e-value $\leq $ 1.0). Usually, reads with high conservation against some RdRp references will be connected to $G_1$. Then, we run DIAMOND BLASTP (e-value $\leq $ 1.0) again to perform all-against-all alignment on all reads. Some of the poorly conserved reads can be connected with the highly conserved reads based on the reads’ similarities. If the input data are too large to use DIAMOND BLASTP efficiently, we resort to String Graph Assembler (SGA) [24] to build the edges between reads based on theirs overlaps. The speed of SGA is much faster than DIAMOND. But because it uses more stringent criteria for read comparison, the accuracy is slightly reduced.

After building $G_1$, we apply Probabilistic Relational Neighbor Classifier (PRC) [17] on it to classify these reads. In $G_1$, $P(c|v)$ is the probability of the read $v$ belonging to class $c$ (i.e. an order). PRC will compute $P(c|v)$ as the weighted average of the class probabilities from $v$’s neighboring nodes. Then, the algorithm will iteratively update the probability of each node until it converges or until the maximum number of iterations is reached. The equation of $P(c|v)$ is shown below:

(1) \begin{align*}& P(c|v)=\frac{1}{\sum_{v_j}^{}w(v, v_j)}\sum_{v_j\in D_v}^{}w(v, v_j)*P(c|v_j) \end{align*}

where $D_v$ is the set of neighboring sequences of read $v$ and $w(v, v_j)$ is the weight of the edge between $v$, and $v_j$. Here, we set the $w(v, v_j)$ of all edges to 1. We also tried to define the weights with e-values or similarities, but there was no improvement in the results.

Macskassy et al. [17] have shown that this method is better than traditional label propagation methods, especially when the data set is imbalanced or few nodes have known labels.

**Figure 6:**
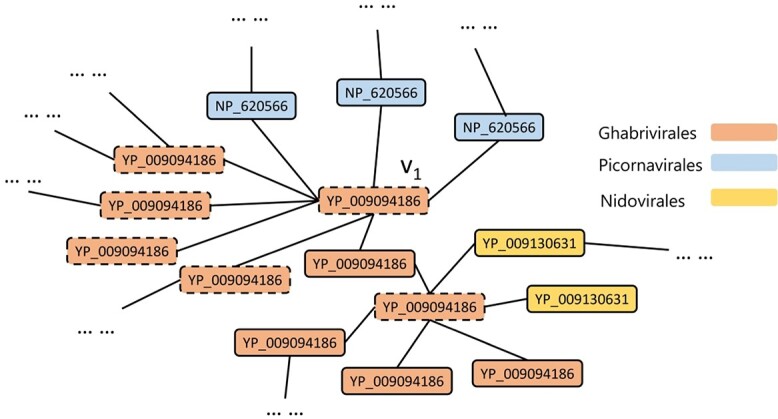
A subgraph built with the simulated RdRp sequences from three orders. The nodes represent translated RdRp reads with a length of 66aa, and these sequences are from *Ghabrivirales*, *Picornavirales* and *Nidovirales*. Nodes with dash lines represent unlabeled reads, while nodes with solid lines are from reference sequences or sequences that can be labeled by BLASTP. Different colors represent different orders of the sequences. The NCBI ID of the sequences is also shown inside the node.

Our experimental results showed that PRC is able to assign correct labels for nodes even if the nodes are connected to ‘wrong’ nodes initially. Figure 6 gave a true example, where the node denoting read $v_1$ is from the order *Ghabrivirales* and is connected to RdRps from more than one order. If we simply apply majority vote, which is a commonly adopted strategy for comparison-based classification, the label of $v_1$ would be assigned with *Picornavirales*. But if we use PRC, $v_1$ will be assigned with the correct order *Picornavirales* after multiple iterations because the probability of $v_1$ being classified into *Picornavirales* increases when its neighboring nodes are classified as *Picornavirales* during the label propagation.

#### Step 2: using motif GCN to classify reads from diverged RdRp genes

To further improve the read classification sensitivity, we will expand $G_1$ by adding motifs as the intermediates. This idea was inspired by text GCN [32], which uses the relationship between words and documents to build graphs and then uses GCN to classify documents. In this task, we will apply motif GCN and contact node classification in the expanded graph. Next, we first detail the graph construction and then describe the graph convolution-based node classification.

***Edge construction in the knowledge graph*  $G_2$**

In the first step, we run HMMER [7] to detect putative RdRp reads from the unlabeled reads. For these putative RdRp reads, if they can match a motif derived from RdRps, we will create new nodes for these reads in the knowledge graph. We will also create the edges between references by running all-against-all DIAMOND BLASTP if e-value $\leq $ 1.

To obtain the motifs of the sequences under different orders, we use MEME [2] to calculate motifs and then use FIMO [9] to search if query sequences contain the derived motifs. The *P*-value of FIMO is used to determine whether the motif is present in a read. We create an edge between a motif and a read if the motif matches the read with *P*-value $\leq 1e-5$.

Then, following the same idea from text GCN, we create edges between motifs according to the point-wise mutual information (PMI). PMI is widely used to measure the weights between words in natural language processing problems and has been proven to enhance the learning ability of GCN [32]. In our formulation, words are motifs and we define the PMI between motif $m_i$ and motif $m_j$ as follows:

(2) \begin{align*}& PMI(i,j)=log\frac{p(i,j)}{p(i)p(j)}, \nonumber\\ p(i,j)&=\frac{\#W(i,j)}{\#W}, \nonumber\\ p(i)&=\frac{\#W(i)}{\#W}, \end{align*}

where $\#W(i)$ is the number of sequences that contain motif $m_i$, $\#W(i, j)$ is the number of sequences that contain motif $m_i$ and motif $m_j$ and $\#W$ is the total number of sequences.

**Figure 7:**
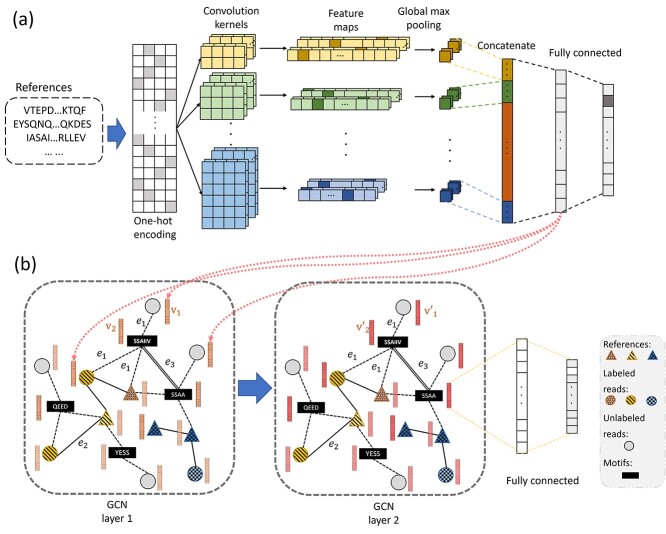
The structure of the embedding model and the text GCN model. **(A)** is the architecture of the CNN model, which contains a convolutional layer with kernels of different sizes followed by a global maximum pooling layer. The outputs of all pooling layers are concatenated and then fed to two fully connected layers. **(B)** is the structure of the GCN model, which contains two graph convolution layers, and the output of the second graph convolution layer is followed by two fully connected layers. In the graph inside **(B)**, e1 indicates the edge between motifs and sequences, e2 represents the edge between sequences and e3 represents the edge between motifs. And we apply the Adam optimizer and use the cross-entropy as the loss function for both models.

Finally, the edges of $G_2$ consist of reads–motif edges, motif–motif edge and reads–reads BLASTP edge. The graph is shown in Figure 7 and the edge weight is defined as follow:

(3) \begin{align*} Weight(m_i, m_j) = PMI(m_i, m_j),\quad &if\ \ PMI(m_i, m_j)> 0, \nonumber\\ Weight(v, m) = 1,\quad & if\ \ p-value(v, m) < 1e-5, \nonumber\\ Weight(v_i, v_j) = 1,\quad & if\ \ e-value(v_i, v_j) < 1, \end{align*}

where $m$ is the motif and $v$ is the read.

***Node representation in the knowledge graph*  $G_2$**

Since the input to the convolution must be numerical vectors, we need to represent all the nodes as numerical vectors. We use the convolutional neural network (CNN) to generate the embedding vector for each node. The network structure is shown in Figure 7. After training the CNN , we fix the model’s weights and then use the output of the penultimate fully connected layer as the embedding feature vectors $v$ for nodes. And we define the feature vectors of a motifs $u$ as follow:

(4) \begin{align*}& u_e=\frac{1}{|N_e|}\sum_{i\in N_e}^{}v_i \end{align*}

where $N_e$ is the set of neighboring nodes of node $e$.

**GCN training**

After constructing the knowledge graph $G_2$ , we apply convolution on the graph to classify the unlabeled reads. GCN can embed the features of neighboring nodes into the target nodes while combining the topological structure of the graph. After applying multiple layers of convolution in $G_2$, the information can be passed from a node to its non-directly connected neighbors. Let the number of nodes in $G_2$ be $n$, and we can represent the graph using the adjacency matrix $A$. Thus, the degree matrix can be represented as $D_{ii} =\sum _{i}^{}A_{ij}$. The formula of the graph convolution layer is listed as follows:

(5) \begin{align*}& H^{l+1}=\sigma (\widetilde{D}^{-\frac{1}{2}}\widetilde{A}\widetilde{D}^{-\frac{1}{2}}H^{(l)}W^{(l)}), \end{align*}

where $H^{(l)}$ is the output from the previous layer and W is the weight matrix. The size of the input feature vector $H^{(0)}$ is $n*l$, where $l$ is the length of the feature. The GCN model is shown in Figure 7  **(B)**. In the training step, all the reference sequences and labeled reads will be used as training samples. In the backpropagation, we update the parameters accordingly and the label information will be delivered from labeled nodes to the unlabeled nodes.

## Experiment

We tested RdRpBin using three types of datasets. First, we evaluated RdRpBin on simulated RNA viral sequencing data, which contains reads sampled from viruses in different orders. For the simulated data with known ground truth, we focus on testing three main factors that can affect the performance of read classification. They are the similarity between the references and the test set, the size ratio of the training to the test set and the sequencing coverage of samples in test set. Then, we tested RdRpBin on a simulated viral marine metagenomic dataset that contains eukaryote, prokaryote, RNA viruses and DNA viruses from the marine ecosystem. Although there are publicly available mock metagenomic datasets, they mainly contain bacteria and thus cannot be conveniently applied to our test. We constructed the simulated metagenomic data by including many non-RNA virus reads from a comprehensive marine genome survey [20]. Finally, we tested RdRpBin on a real metagenomic data.

For all the experiments, we benchmarked RdRpBin against several popular and extensively tested read-level taxonomic classification tools that can be applied to RNA viruses, including DIAMOND, MMseqs2, Kaiju and Kraken2.

### Read classification for simulated RNA viral sequencing data

We simulated the reads from the NCBI-RdRp dataset, which contains the RdRp sequences identified from the NCBI database (see Section 2.1 for its construction). As shown in Figure 2, not every order has abundant training data. Thus, the performance evaluation is divided into two parts: one is for all the 18 orders, and the other is for the three most abundant orders. The latter can reflect the ability of learning more accurately. We constructed eight pairs of training and testing RdRps ($D_{train}$ and $D_{test}$) by configuring the three factors.

**Similarity between $D_{train}$ and $D_{test}$**. High divergence between $D_{train}$ and $D_{test}$ poses a challenge for both alignment-based and learning-based read classification. In order to create $D_{train}$ and $D_{test}$ with controllable similarities, we apply CD-HIT to cluster the RdRp sequences in the NCBI-RdRp dataset using $\tau $ as the threshold. Then, for each cluster, only the longest sequences from each cluster are kept. Therefore, the similarity between all kept RdRps will be less than the threshold $\tau $. Then, we randomly divided the data into $D_{train}$ and $D_{test}$, where the similarity between $D_{train}$ and $D_{test}$ will be less than $\tau $. In the experiment, we tested two similarity cutoffs: 0.4 and 0.6.

**Ratio of $D_{train}$ and $D_{test}$**. Small training data (i.e. number of reference sequences) can affect the performance of both learning and alignment-based tools. We split all the RdRps extracted by CD-HIT into $D_{train}$ and $D_{test}$ with two ratios: 1:2 and 2:1.

**Coverage of samples in test set**. Because we use the similarity or overlaps between reads to build edges, the coverage of the sequencing reads will influence the connectivity of the graph. To evaluate the performance of RdRpBin on data with different coverage, we generated test sets with coverage of 1X and 5X, respectively.

Table 1 and Table 2 show the number of training and testing RdRps and their generated training and test sets in the 18-order and the three most abundant-order experiments, respectively.

**Table 1:** The properties and sizes of $D_{train}$ and $D_{test}$ in the 18-order experiment^*^.

Sim	Ratio	Cvge	$\boldsymbol{|D}_{\boldsymbol{train}}\boldsymbol{|}$	$\boldsymbol{|D}_{\boldsymbol{test}}\boldsymbol{|}$	# of training samples	# of testing samples
0.4	1: 2	1X	111	221	4302	1775
0.4	1: 2	5X	111	221	4302	8990
0.4	2: 1	1X	221	111	9014	865
0.4	2: 1	5X	221	111	9014	4350
0.6	1: 2	1X	297	593	12 409	4699
0.6	1: 2	5X	297	593	12 409	23 827
0.6	2: 1	1X	593	297	24 773	2289
0.6	2: 1	5X	593	297	24 773	11 561

^*^Note that the number of sequences is smaller than Figure 1 because of clustering by CD-HIT. Sim: the similarity between $D_{train}$ and $D_{test}$. Ratio: $\frac{|D_{train}|}{|D_{test}|}$. Cvge: the coverage of the testing samples.$|D_{train}|$: the number of training RdRps.$|D_{test}|$: the number of testing RdRps. Training and testing samples: reads from RdRp genes.

**Table 2:** The properties and sizes of $D_{train}$ and $D_{test}$ in the three most abundant orders.

Sim	Ratio	Cvge	$\boldsymbol{|D}_{\boldsymbol{train}}\boldsymbol{|}$	$\boldsymbol{|D}_{\boldsymbol{test}}\boldsymbol{|}$	# of training samples	# of testing samples
0.4	1: 2	1X	50	101	2476	1009
0.4	1: 2	5X	50	101	2476	5307
0.4	2: 1	1X	101	50	5320	494
0.4	2: 1	5X	101	50	5320	2592
0.6	1: 2	1X	119	238	7061	2463
0.6	1: 2	5X	119	238	7061	13 026
0.6	2: 1	1X	238	119	13 726	1200
0.6	2: 1	5X	238	119	13 726	6339

Compared with the experiment in Table 1, the experiment in Table 2 contains only the three most abundant orders, so the number of samples is smaller than that in Table 1.

**Figure 8:**
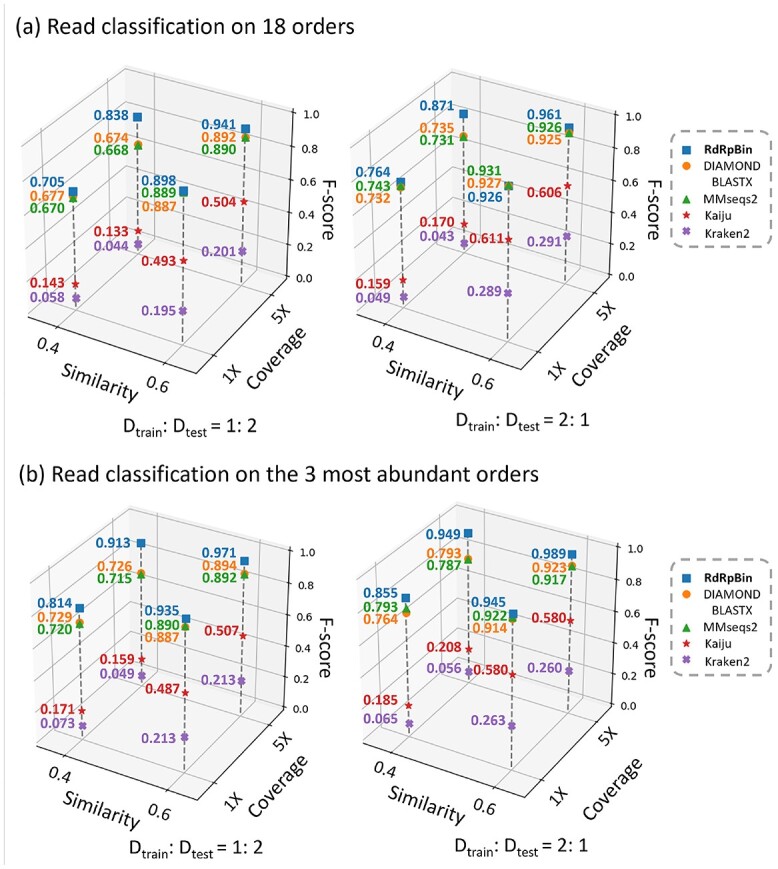
Classification performance using simulated data: **(A)** is the performance on 18 orders and **(B)** is the performance on the three most abundant orders.

We use ART [11] to simulate the test set with different coverage. We compared RdRpBin with DIAMOND BLASTX, MMseqs2, Kaiju and Kraken2 on 18-order and the three most abundant-order datasets. The results are shown in Figure 8. DIAMOND BLASTX translates the query reads into proteins and aligns these proteins to the reference database. Then, we use the majority vote to get the taxa. MMseqs2 uses $k$-mer matching and vectorized ungapped and gapped alignment for sequence annotation. Kaiju is a taxonomic classification tool that uses the maximum exact match to find matching sequences in protein reference databases. Kraken2 conducts taxonomic classification for metagenomic sequencing data based on $k$-mer matching and lowest common ancestor voting. We run all these tools using their default parameters.

As shown in Figure 8, the tools sorted by the descending order of the average F-score are RdRpBin, DIAMOND BLASTX, MMseqs2, Kaiju and Kraken2. The results show that similarity plays an important role on the classification performance. The tools that rely on exact match, including Kaiju and Kraken2, have the average F-score reduction of 72.7% and 80.1%, respectively, when the similarity decreases from 0.6 to 0.4. In contrast, when the similarity is 0.4, RdRpBin still has a high F-score. In addition, its average F-score is 12.8% higher than that of the second-best tool DIAMOND BLASTX. Although the F-score of all tools decreases as the training set becomes smaller, RdRpBin also has the best performance on a small training set. When $D_{train}: D_{test}$ is 2: 1 and 1: 2, RdRpBin’s average F-score is 5% and 7% higher than that of DIAMOND BLASTX, respectively. Moreover, as expected, The average F-score of RdRpBin is much higher than other tools when the coverage increases. The reason is that more edges can be built between reads, and using PRC can lead to a higher recall. The average F-score of RdRpBin is 2.2% and 11.9% higher than DIAMOND when the coverage is 1X and 5X, respectively. When we focus on the three most abundant orders (*Bunyavirales*, *Mononegavirales*, *Picornavirales*), all tools has better performance as shown in Figure 8  **(B)**. And RdRpBin still has the best F-score.

**Identification of unknown taxa and classification at lower taxonomic ranks.** To accommodate new viruses that are not part of the trained labels of RdRpBin, we allow RdRpBin to identify those reads as ‘others’. The detailed method and the experimental results can be found in Supplementary Section 1. In addition, RdRpBin can be conveniently applied to other taxonomic ranks. However, the limited training samples can affect its generality at lower taxonomic ranks. We demonstrate the utility of RdRpBin at family and genus levels in Supplementary Section 2.

### Experiment on simulated marine metagenomic data

While the previous experiment focuses on evaluating the classification performance using pure RNA viruses as input, the experiment in this section will use simulated marine metagenomic data as input. Despite the advances in viral metagenomic sequencing protocols, the real viral metagenomic data are still loaded with contamination from eukaryotes and prokaryotes, etc. In order to test the read classification tools in a more realistic scenario, we created a simulated marine metagenomic dataset that mixes RNA viruses with other species. We choose the marine ecosystem because it is a habitat of a large number of viruses. To cover a broad range of marine organisms, we created the dataset using two sources, both of which cover many different marine species. It is also worth noting that transcriptomic sequencing is needed to sequence RNA viruses. In this simulated data set, we used the whole genomes for simulating reads, which cover all genes and thus will not reduce the difficulty of read classification.

The first source is based on the marine organisms in the World Register of Marine Species (WoRMS) [10]. We extracted the list of the marine RNA viruses and marine DNA viruses. Among these viruses, *Bunyavirales*, *Mononegavirales* and *Picornavirales* are very abundant in marine. Thus, we chose all 16 RNA viruses from these three orders and all 47 DNA viruses to simulate viral reads. We employed CAMISIM [8] to generate simulated data. CAMISIM can generate simulated data that have high functional congruence to the real data. We simulated the metagenomic data with the relative abundance following a log-normal distribution, whose mean and standard deviation were set to 1 and 2, respectively. And we used ART as the engine of CAMISIM to simulate Illumina 200 bp reads. Finally, the simulated data contain 31 382 RNA virus reads and 131 717 DNA virus reads.

We created the second part by adding simulated reads from eukaryotes and prokaryotes in the marine ecosystem. We randomly sampled reads from a simulated marine metagenome (NCBI SRA ID: ERR2185279), which was created by Alex et al. [20] and contained 12 500 000 pairs of 250 bp reads. These data simulate the high diversity of organisms in marine. They contain the reads from 82 eukaryotic, 365 prokaryotic and DNA/ RNA viruses. Moreover, their sequencing error profile was generated from real data, and shuffled reads were also added as unknown reads. To build the second part of the dataset, we randomly extracted 1500 000 non-virus reads from these simulated data and kept only the first 200 bp of each read because we currently used the vectors of length 200 in the CNN. In practical applications, the vector size can be adjusted based on the most commonly seen read size. Finally, we combined these two datasets and got the simulated metagenomic dataset.

To focus on the difficult case where the test set has diverged from the reference sequences, we removed the reference viruses that have similarity with the test set above 40%. We aligned RdRps from 16 RNA viruses to the NCBI-RdRp dataset by DIAMOND BLASTP and removed all reference RdRps with similarity greater than 0.4 in NCBI-RdRp. Finally, the numbers of reference RdRps under *Bunyavirales*, *Mononegavirales* and *Picornavirales* are 34, 40, and 60, respectively. All the benchmarking tools used this reference database and were run using their default parameters.

**Figure 9:**
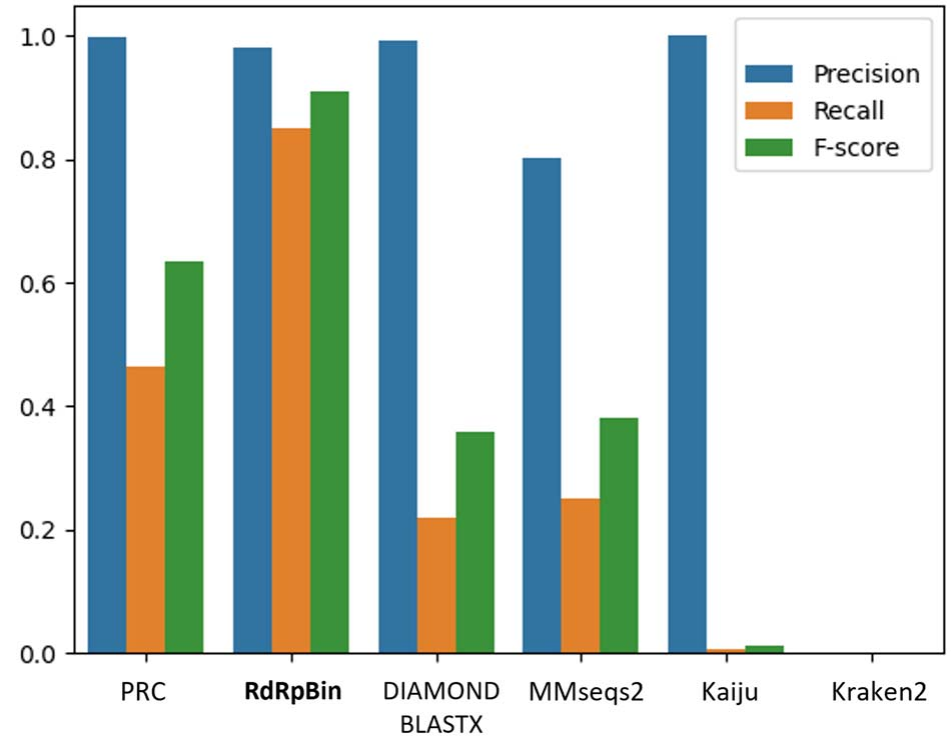
Benchmarking results on the simulated marine metagenomic data.

Because the reference sequences only contain RdRps, we treat all the RdRp reads as the true samples and all the other reads as false, including the reads from other regions of RNA viruses. Because we have the RdRp annotations for the 16 RNA viruses, we can decide the reads’ origins using BLASTN with near 100% identity. Figure 9 summarized the read classification results of all benchmarked tools on this dataset. The performance of Kaiju and Kraken is not good under the default parameters. Although the precision of Kaiju is 1, its recall is only about 0.007. And Kraken2 does not identify any reads in the test set. DIAMOND BLASTX and MMseqs2 are both alignment-based tools. Although MMseqs2 has lower precision, it has a higher recall (0.251) than DIAMOND BLASTX (0.218), leading to a slightly better F-score. Using PRC can increase the recall to 0.464 while keeping the precision at 0.997, compared with BLASTX. The RdRpBin can further improve the recall to 0.849.

The contamination from eukaryotes and prokaryotes can affect viral composition analysis. There are some tools available to estimate the level of viral enrichment, such as ViromeQC [34]. We conducted a series of experiments using this tool and RdRpBin on different simulation datasets. The results can be found in Supplementary Section 3.

### Experiment on the real metagenomic data

We used real metagenomic data to test the performance of our method and other tools. The dataset was downloaded from NCBI SRA (id: SRR9216246). Gideon et al. [21] sampled these data from Oncorhynchus tshawytscha and discovered a new coronavirus called Pacific salmon Nidovirus, which belongs to *Nidovirales*. These data may also contain some other RNA viruses.

**Figure 10:**
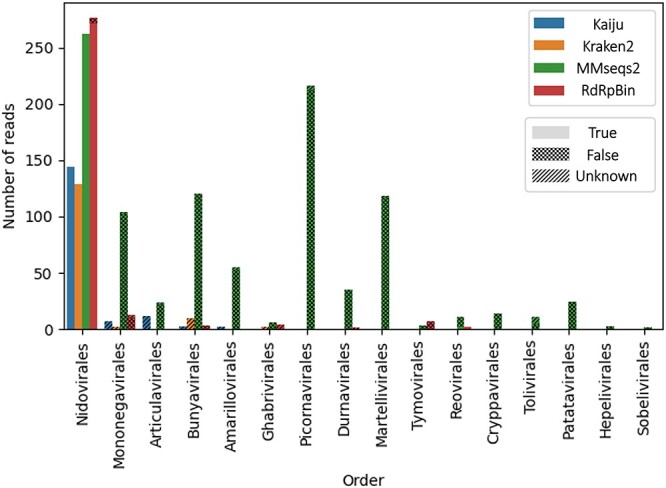
The number of RdRps found under different orders with different tools. True: RdRp reads supported by BLAST. False: misclassified reads. Unknown: reads that have no known origin. MMseqs2 has many misclassified reads for multiple orders.

During pre-possessing, we used fastp [5] to filter the low-quality reads with its default parameter. If more than 40% of the bases in the reads have a quality value below Q15, or the reads contain more than five Ns, we remove these reads. And we used Bowtie 2 [13] to filter out the reads belonging to the host. After pre-processing, 14 031 851 reads were left. To construct a relatively comprehensive RdRp reference dataset, we used the representative sequences extracted using CD-HIT with a threshold of 0.95 from the NCBI-RdRp database. This reference database contained 18 orders and 7008 RdRps. We compared RdRpBin with MMseqs2, Kaiju and Kraken2. We did not compare with DIAMOND BLASTX because we used BLAST to determine the ground truth of reads, which was not fair for other tools when calculating the precision. All the tools used this dataset as the reference database and were run using their default parameters. The final results are shown in Figure 10.

We then examined whether the identified reads are RdRp reads by aligning them to NCBI-RdRp database using BLASTN with near 100% identity. Based on the results, we divided the identified reads into three parts: true, false and unknown. True refers to reads that are aligned to the annotated RdRp genes in the corresponding orders. False means that the reads are not aligned to RdRp genes or aligned to the wrong orders. And unknown reads mean that the reads cannot be aligned to any reference sequence. The number of aligned RdRp reads and the precision of each method on the real data are shown in Figure 10 and Table 3, respectively. We can see that although MMseqs2 classifies the most reads, it tends to overestimate the reads in each order and its precision is not high. Although Kaiju and Kraken2 have high precision and fast speed, the number of identified RdRp reads was low ($\leq $150). Our tool achieves a superior trade-off between recall and precision.

**Table 3:** The precision of each method on the real data.

Method	# of found RdRp reads	# of aligned RdRp reads by BLASTN	Precision	Running time (min)$^a$
RdRpBin$^b$	306	285	0.931	32.2
Kaiju	165	165	1.0	3.2
Kraken2	142	142	1.0	0.5
MMseqs2	1005	270	0.269	26.4

${}^{a}$ Benchmarking was performed on a PC with Intel Core i7-9700 (8 cores) CPU and GeForce RTX 2070 GPU.${}^{b}$SGA was used to build edges between reads. If the overlap between reads was bigger than 80 bp, the edge was built.

### Discussion

RNA viruses have high diversity. RdRp is the only gene possessed by most RNA viruses. By employing RdRp and graph-based read classification method, we developed RdRpBin to improve the trade-off between recall and precision for RNA virus read classification, which helps derive RNA virus composition and abundance profiles from heterogeneous data. In particular, when the similarity between the test RNA viruses and the reference database is low (such as 0.4), RdRpBin can still maintain a high F-score (>0.8).

The advantages of using GCN are evident for large orders. However, for small classes, lacking sufficient training data jeopardizes the learning ability of GCN. In addition, the number of RdRps in different orders varies significantly. This type of imbalance, if not addressed carefully, can affect the performance of learning. Reads from rare orders tend to be classified into big orders. Data imbalance is a well-studied problem in the field of machine learning. However, conventional methods such as data oversampling and undersampling do not work well for genomic data. We will employ some recently developed methods such as logit adjustment to address this challenge in our future work.

The two most time-consuming steps of RdRpBin are building edges between reads and identifying potential RdRp reads using HMMER, which take $\sim 30\%$ and $\sim 25\%$ of the running time in the real data experiment, respectively. For the former, faster reads similarity measuring tools can help RdRpBin reduce running time. For the latter, we will try more efficient homologous sequence search methods in the future.

**Key Points**

- RNA virus taxonomy classification is a key step for viral composition analysis and novel RNA virus discovery in metagenomic data.
- We developed a read classification tool named RdRpBin that uses RNA-dependent RNA polymerase gene for RNA virus composition analysis.
- By combining alignment-based strategies and graph-based learning models, RdRpBin outperformed other state-of-the-art tools on both simulated and real sequencing data.

## Supplementary Material

suppl_data_bbac011

## Data Availability

RdRpBin is implemented in Python, which can be downloaded at https://github.com/HubertTang/RdRpBin.

## Notes

**Xubo Tang** is currently pursuing his Ph.D. degree at the City University of Hong Kong. His main research interests include computational biology and bioinformatics, particularly applying deep learning models to analyze genomic data.

**Jiayu Shang** received his bachelor’s degree from Sun Yat-sen University. He is now pursuing his Ph.D. degree at the City University of Hong Kong. His research interest is bioinformatics, with a focus on algorithm design for analyzing microbial sequencing data.

**Yanni Sun** is currently an associate professor in the Department of Electrical Engineering at the City University of Hong Kong. She got her Ph.D. in Computer Science and Engineering from Washington University in Saint Louis, USA. Her research interests are sequence analysis and metagenomics.
