# Supplementary material for: RdRp-based sensitive taxonomic classification of RNA viruses for metagenomic data
Source: Brief Bioinform. 2022 Feb 7;23(2):bbac011. doi: 10.1093/bib/bbac011 (PMC8921650; doi:10.1093/bib/bbac011)
Supplement: suppl_data_bbac011 [file suppl_data_bbac011.zip › SUPPLEMENTARY.pdf]

SUPPLEMENTARY

1. Experiment on classification with unknown taxa

The classification is often an open-set problem in real applications, where the input data can contain unknown classes that do not appear in the training dataset. So, we evaluate whether RdRpBin can identify the reads from unknown taxa. Because reads from unknown taxa cannot be mapped to the reference genomes (reference nodes in Graph 1 of Figure 5), the reads from unknown taxa will form a subgraph rather than be connected to the graph containing reads of known labels. Although some reads from those known labels cannot connect to the main graph either because they do not share overlaps/similarity/common motifs with other reads, they are often in isolated graphs of very small sizes, even forming singletons (single-node graph). Thus, we will assign reads in the isolated subgraphs as "other" if the size of the subgraph is larger than a pre-set threshold.

We used reads that are not in our 18 orders to test this method. These reads are from *Jingchuvirales* and *Mindivirales*, which are the "new" taxa. We tested whether our model could correctly assign "other" label to them. These two orders are very small, so they were not included in our reference database of RdRpBin. We built the test dataset by mixing a set of data (the similarity is 0.6, the ratio is 2: 1, and the coverage is 5X) D_{known} from Section "Read classification for simulated RNA viral sequencing data" with the simulated unknown taxa data $D_{unknown}$. $D_{unknown}$ contains 4 RdRps from *Jingchuvirales* and 14 from *Mindivirales*. We used ART to simulate the reads in $D_{unknown}$ with a coverage of 5X. The reference contains 593 RdRps, and the test set contains 9361 simulated reads, of which 8510 are from D_{known} and 851 are from $D_{unknown}$. Assuming that reads of length 200bp are uniformly distributed over RdRp of length greater than 1500bp, a coverage is 5X requires at least 37.5 reads. Thus, we set the graph size threshold to 37, and any isolated subgraph larger than this threshold will

be given the label “other”. We used recall and precision to evaluate the performance, and the formula is shown below:

$$Recall = \frac{the\ number\ of\ identified\ "other"\ reads}{the\ number\ of\ total\ "other"\ reads}$$

$$Precision = \frac{the\ number\ of\ identified\ "other"\ reads}{the\ number\ of\ identified\ reads}$$

The recall and precision of the “other” label are 0.69 and 1.00, respectively. Some “other” reads subgraphs are too small to be identified, which leads to a lower recall. Among these identified subgraphs, any subgraph containing reads from the “new” order is always pure. Thus, assigning the whole subgraph as “other” is correct for the unknown orders. In addition, the results for the other 18 orders (i.e., “known” labels) are the same as Fig. 8 in the paper.

2. Experiment on the lower taxonomic levels

To evaluate RdRpBin at the lower taxonomic levels (family and genus), we use *Picornavirales* because it is the most diverse order in our dataset. We show the family-level classification performance for the families under *Picornavirales* and genus-level classification performance under each family. For each family and genus, we use CD-HIT to remove the redundant sequences with a similarity cutoff of 0.8. And we only keep the families that contain more than one genus and the genera that contain at least 5 RdRps. The number of sequences of each family is shown in Figure 1. The performance of RdRpBin at different taxonomic levels is shown in Table 1.

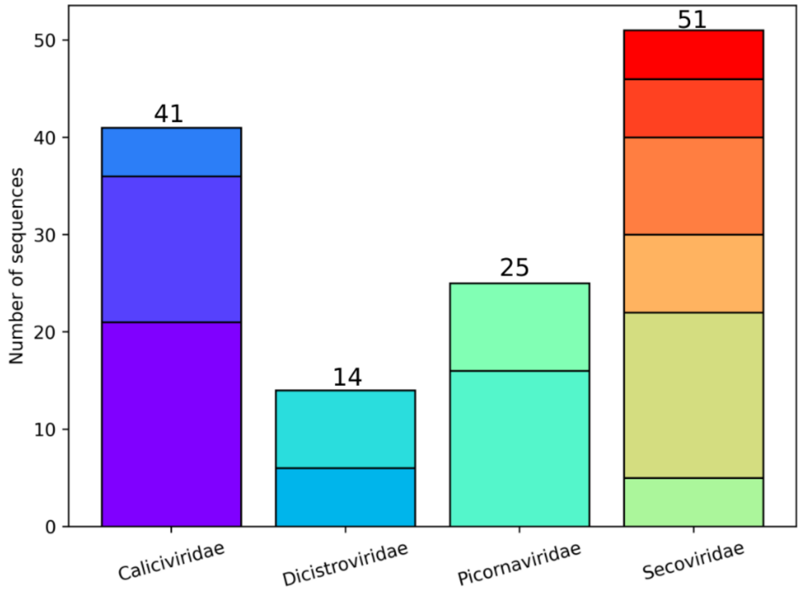

Figure 1. The number of RdRPs in 4 families under *Picornavirales* in the database. X-axis: the names of the 4 families. The blocks with different sizes and colors in the bar represent the genera under the corresponding family.

Table 1. The performance of read classification at the family and genus levels under *Piconavirales*.

Taxon	taxonomic level for classification	# of reference RdRps	# of testing samples	Precision	Recall	F-score	# of classes
Picornavirales	family	168	1273	0.971	0.970	0.970	4
Caliciviridae	genus	29	345	0.884	0.849	0.844	3
Dicistroviridae	genus	10	64	0.894	0.867	0.863	2
Picornaviridae	genus	17	116	0.994	0.994	0.994	2
Secoviridae	genus	37	322	0.926	0.898	0.896	6

Based on Table 1, we can see that the F-score at the family level is 0.97, and the average F-score at the genus level is 0.899. And the F-scores at the family and genus levels are smaller than that at the order level, which is 0.983 for *Piconavirales*. At the genus level, the F-score decreases because there are not enough references. For example, the family Dicistroviridae has 2 genera, containing only 6 and 4 RdRps respectively

as references. With sufficient training samples at lower ranks, RdRpBin can be conveniently applied to lower ranks.

3. Experiment on the simulated data with different levels of viral enrichment

The contamination from eukaryotes and prokaryotes can affect viral composition analysis. To evaluate the performance of RdRpBin under different viral enrichment, we used the data mentioned in Section "Experiment on simulated marine metagenomic data" to build the test dataset. We sampled reads from ERR2185279 and the simulated viral metagenomic data and combined them using different ratios. We then used ViromeQC [1] to estimate their viral enrichment. ViromeQC can provide a viral enrichment score for metagenomic datasets based on rRNA and bacterial marker alignment. We then ran RdRpBin on the test dataset to evaluate the performance. The ViromeQC score and result are shown in Table 2.

Table 2. The performance of RdRpBin in simulated data with different enrichment scores.

Sample ID	ViromeQC score	Precision	Recall	F-score
1	1.5X	0.997	0.834	0.908
2	4.4X	0.998	0.833	0.908
3	8.4X	0.998	0.836	0.910
4	12.2X	0.999	0.844	0.915

We can see that the F-score of RdRpBin increased from 0.908 to 0.915 as the enrichment of the virus increased from 1.5X to 12.2X. We can observe a positive correlation between the ViromeQC score and classification performance. The reduction of contaminants leads to an increase in precision. And a higher abundance of viral reads ensures that RdRpBin can classify more reads by building graphs, which increases the recall. For ViromeQC, it is considered that the viral enrichment of the data is high

when the score is greater than 10. RdRpBin can still achieve an F-score of over 0.9 even when the ViromeQC score is 1.5X.

References

[1] Zolfo, M., Pinto, F., Asnicar, F., Manghi, P., Tett, A., Bushman, F. D., & Segata, N. (2019). Detecting contamination in viromes using ViromeQC. Nature Biotechnology, 37(12), 1408-1412.
