# Supplementary material for: RdRp-based sensitive taxonomic classification of RNA viruses for metagenomic data
Source: Brief Bioinform. 2022 Feb 7;23(2):bbac011. doi: 10.1093/bib/bbac011 (PMC8921650; doi:10.1093/bib/bbac011)
Supplement: suppl_data_bbac011 [file Response Letter.pdf › Response Letter.pdf]

Dr. Yanni Sun (the corresponding author)
Dept. of Electrical Engineering
City University of Hong Kong
Hong Kong (SAR)
yannisun@cityu.edu.hk

Dear Editors and Reviewers:

We would like to thank the editor for the opportunity to revise the paper and also the reviewers for the valuable comments. This letter describes the detailed response to each comment and the corresponding modifications to our manuscript submitted to Brief in Bioinformatics (Manuscript ID: BIB-21-1678). All the changes are highlighted in red in the manuscript. Before providing the detailed responses, we summarize the major modifications below.

1. We explained how RdRpBin identifies reads from “unknown” taxa and added experimental results for this utility.
2. We tested the suggested viral enrichment estimation tool ViromeQC on multiple simulation datasets.
3. We added the classification performance of RdRpBin at other taxonomic ranks.

Because of the 5000 word limit, we added the new results to the supplementary file. We hope that the following point-to-point responses will be clear enough to convey our ideas and address the comments of the reviewers. All the comments are in italic, and our responses are in blue.

**Comments of Reviewer #1:**

*Xubo Tang et al., submitted a manuscript for consideration of publication in Briefings in Bioinformatics, which was entitled as “RdRp-based sensitive taxonomic classification of RNA viruses for metagenomic data”. In the manuscript, the authors designed a RNA virus classification tool RdRpBin, which is based on sequences of RdRp gene-based reads. At first, they used the RdRp reference database to cluster the sequence patterns at order level, calculate the frequencies of the aligned sites in RdRp sequences and define the well-conserved sites in RdRp genes in each order. In this section, they used String Graph Assembler to construct the edges between the partially overlapped reads and calculated their divergences, then used neighbor-joining relationship between the unlabeled and the well-defined reads, and constructed the taxonomic networks in the metagenomic analysis. Second, they utilized the frequencies of motifs in the RdRp sequences of each order to define the taxonomic position of divergent reads in the metagenomic datasets. The authors’ works are interesting and essential for classification of RNA viruses in areas of metagenomic analysis, as there are not quite well-established databases for the metagenomic analysis of RNA viruses.*

***Comment 1****. How do the authors control the qualities of the reads for metagenomic analysis? There are some sequencing errors and chimeric reads in the metagenomic analysis of RNA viruses, and those chimeric reads have an impact on defining the new virus.*

**Response**: We used fastp [1] for read quality control. In this revision, we added more details about the preprocessing step. Based on the default parameters of fastp, if more than 40% of the bases in the reads have a quality value below Q15, or the reads contain more than 5 N bases, we remove these reads. We revised the manuscript accordingly in Section "Experiment on the real metagenomic data", paragraph 2.

For chimeric reads, a survey [2] shows that chimerism is more likely to occur between reads with microhomology, and intra-genome chimerism is more frequent than chimerism between genomes. Given that we classify reads at the order level, the chimeric reads from the same species do not have a significant impact on RdRpBin. But if there are many chimeric reads from RdRp region, it is possible that RdRpBin will classify them as "unknown" taxa. For our simulated data, there are no chimeric reads. For the real metagenomic data with a known RNA virus, we mapped the reads back to the reference genome and did not find such cases either.

**Reference**:

[1] Chen, S., Zhou, Y., Chen, Y., & Gu, J. (2018). fastp: an ultra-fast all-in-one FASTQ preprocessor. Bioinformatics, 34(17), i884-i890.

[2] Peccoud, J., Lequime, S., Moltini-Conclois, I., Giraud, I., Lambrechts, L., & Gilbert, C. (2018). A survey of virus recombination uncovers canonical features of artificial chimeras generated during deep sequencing library preparation. G3: Genes, Genomes, Genetics, 8(4), 1129-1138.

***Comment 2**. The authors designed a protein-translation tool for the users' works, which can generate six reading frames. It is a good point. However, as far as I am concerned, the authors should take it in further consideration and design an advanced orf-finder tool and filter out the longest ORF in the datasets.*

**Response**: We agree with the reviewer, and we indeed only keep the longest translations. We clarified this process in the revised manuscript.

Because RdRpBin is developed to classify RNA viruses at the read level, the translation is conducted for reads. Given that reads are short, we remove all translations containing stop codons. This is equivalent to keeping the longest possible translations for a read. This method of only keeping the translations without stop codons works well for most reads that are sequenced from inside RdRps. However, a small number of reads sequenced from the end region of the gene may get discarded because of the stop codon. Therefore, in this revision, we have added an ORF length-related parameter to the program. The user can set a threshold for the length of the translated amino acid sequence. All translations larger than the threshold will be retained. The changes in the manuscript can be found in Section "Overview of the algorithm", paragraph 3.

***Comment 3**. Here is a typo mistake 'well-served' in line 53 in page 3. I think it should be 'well-conserved', not 'well-served'.*

Response: Thanks for pointing this out. We have corrected it.

**Comments of Reviewer #2:**

*The authors developed a new method for the taxonomic classification of RNA viruses, which is helpful for the field.*

***Comment 1**. How to deal with the unknown taxon? Is it possible to add one "other" class?*

**Response**: Thanks for the suggestion. It is useful to have one "other" label for any read from a new or unknown taxon. We thus added this function to RdRpBin in this revision. Because reads from unknown taxa cannot be mapped to the reference genomes (reference nodes in Graph 1 of Figure 5), the reads from unknown taxa will form a subgraph rather than be connected to the graph containing reads of known labels. Although some reads from those known labels cannot connect to the main graph either because they do not share overlaps/similarity/common motifs with other reads, they are often in isolated graphs of very small sizes, even forming singletons (single-node graph). Thus, we will assign reads in the isolated subgraphs as "other" if the size of the subgraph is larger than a pre-set threshold.

Then, we added a new experiment to evaluate the performance of this method. We used reads that are not in our 18 orders to test this method. These reads are from *Jingchuvirales* and *Mindivirales*, which are the "new" taxa. These two orders are very small, so they were not included in our reference database of RdRpBin. We tested whether our model could correctly assign "other" label to them. We thus built a new testing dataset by combining these "new" taxa reads with the test data from Section "Read classification for simulated RNA viral sequencing data". Assuming that reads of length 200bp are uniformly distributed over RdRp of length greater than 1500bp, a coverage is 5X requires at least 37.5 reads. Thus, we set the graph size threshold to 37, and the result shows that the recall and precision of "other" taxa are 0.69 and 1.00, respectively. Some "other" reads subgraphs are too small to be identified, which leads to a lower recall. It is worth noting that any subgraph containing reads from the "new" order is always pure. Thus, assigning the whole subgraph as "other" is correct for the unknown orders. In addition, the results for the other 18 orders (i.e., "known" labels) are the same as Fig. 8 in the paper. Detailed description and the experimental results can be found in Supplementary "Experiment on classification with unknown taxa".

***Comment 2**. How to deal with the segmented ones? It is also a question even for single-strand viruses under the condition of high throughput or meta sequencing.*

**Response**: For the segmented RNA viruses, our database also contains their segmented RdRps. For example, Influenza virus genome contains eight segments, and our database contains RdRp subunit PB1 (PF00602), RdRp subunit PA (PF00603), and RdRp subunit PB2 (PF00604). So, although the RdRps are segmented, we can still identify them using our database and RdRpBin. We clarified this in Section "Sequence properties of RdRp", paragraph 1. We also have many single-strand viruses in the reference database or in the test datasets. Usually, RNA-Seq is applied

to sequence the RNA viruses. As RdRpBin is applied to reads rather than contigs, we can cope with cases where the reads cannot be assembled into contigs due to low abundance.

**Comments of Reviewer #3:**

*This paper described an RNA virus taxonomic classification tool for metagenomic sequencing data of the RdRp gene. A two-step algorithm was proposed to deal with high conserved regions and less similar regions using alignment-based method and AI based method. The tool called RdRpBin was created. Compared to existing state-of-the-arts tools with similar functions, it has comparable performance in most case and better performance for viruses with low sequence similarity. The paper was well written and the comparison of different tools was comprehensive.*

***Comment 1**. In line 15, page 9, authors mentioned that "the bottleneck of RdRpBin in running time are building edge between reads and identifying potential RdRp reads using HMMER, which take up ~30% and ~25% of the runtime in the real data experiment, respectively.". This should be revised since Table 3 showed that the running time of RdRpBin was 10 times and 64 times as Kaiju and Kraken2 respectively. This meant that other steps of RdRpBin were also much slower than existing methods.*

**Response**: Thank you for the comment. We explained this more carefully in the revised manuscript. We have changed this sentence to "The two most time-consuming steps of RdRpBin are building edges between reads and identifying potential RdRp reads using HMMER, which take ~ 30% and ~ 25% of the running time in the real data experiment, respectively."

Other steps are indeed slower than Kaiju and Kranken2. For example, it takes about 5 minutes to train the GCN, accounting for ~15% of the total time.

***Comment 2**. For the performance on real dataset shown in Fig. 10, blast results were used as the ground true results. It would be better to call "the true RdRp reads" as "RdRp reads supported by blast" since the ground truth sequences are unknown for a real dataset.*

**Response**: Thanks for your suggestions. We corrected the description in the revision.

**Comments to the Reviewer #4:**

*In this paper, Tang et al. presented a new method of classifying RNA viruses from metagenomic reads. The authors performed a solid evaluation of the algorithm, using both simulated and real data, showing that it reaches a better trade-off between recall and precision compared to other available tools. RdRpBin appears to be robust and able to deal with low similarity, which makes it a valuable tool for the discovery of new putative RNA viruses from metagenomic and metatranscriptomic data.*

***Comment 1**. On page 8 (lines 3-6), the authors mention the fact that viral metagenomic data (i.e., viromes) are still affected by contamination from eukaryotes and prokaryotes. A recently developed tool (ViromeQC; Zolfo et al., 2019) allows users to score viromes for their quality (absence of contaminants). It would be interesting to test RdRpBin on different viromes scored with this method to check if its performance varies according to the level of viral enrichment (which indicates the absence/presence of contaminants in relation to metagenomes with no virus enrichment).*

**Response**:

Thank you for bringing ViromeQC [1] to our attention. We tested RdRpBin using simulated data with different ViromeQC scores. We sampled reads from ERR2185279 and the simulated viral metagenomic data used in Section "Experiment on simulated marine metagenomic data" and combined them using different ratios. We ran ViromeQC on these data to evaluate their viral enrichment scores. The ViromeQC score and results are shown in the following table.

The performance of RdRpBin in simulated data with different enrichment scores.

| Sample ID | ViromeQC score | Precision | Recall | F-score |
|---|---|---|---|---|
| 1 | 1.5X | 0.997 | 0.834 | 0.908 |
| 2 | 4.4X | 0.998 | 0.833 | 0.908 |
| 3 | 8.4X | 0.998 | 0.836 | 0.910 |
| 4 | 12.2X | 0.999 | 0.844 | 0.915 |

The result shows that the F-score of RdRpBin increased from 0.908 to 0.915 as the enrichment of the virus increased from 1.5X to 12.2X. We can observe a positive correlation between the ViromeQC score and classification performance. We have added these experimental results to Supplementary "Experiment on the simulated data with different levels of viral enrichment".

**Reference**:

[1] Zolfo, M., Pinto, F., Asnicar, F., Manghi, P., Tett, A., Bushman, F. D., & Segata, N. (2019). Detecting contamination in viromes using ViromeQC. *Nature Biotechnology*, *37*(12), 1408-1412.

***Comment 2****. My major concern regards the lack of information for some viral groups (as pinpointed by the authors in the discussion of the present study), which could affect the training of the Graph Convolutional Network. Eventually, it would be useful to show how well the tool performs at other taxonomic levels.*

**Response**:

Thanks for your comment. We agree that we should test the performance of RdRpBin on different taxonomic levels. The manuscript focused on the taxonomy classification at the order level because it is more stable and contains enough training data to train a good model. In the revision, we evaluate RdRpBin at the lower taxonomic level (family and genus).

Given that *Picornavirales* is the most diverse order in our dataset, we classified the families under *Picornavirales* and then classified the genus under each family. The number of sequences of each family is shown in the following figure. The performance of RdRpBin at different taxonomic levels is shown in the following table.

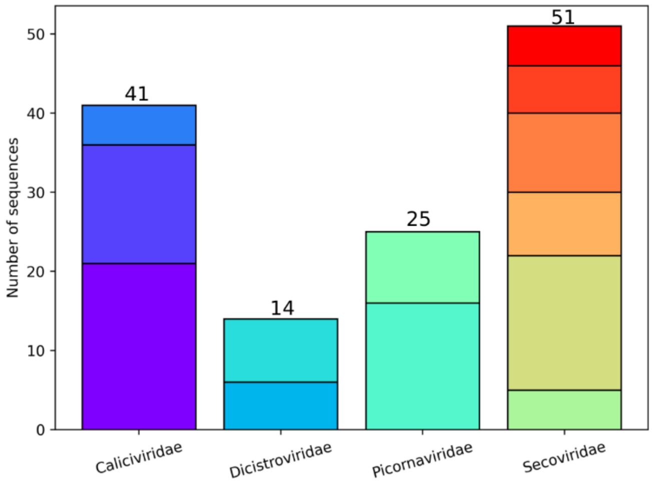


The number of RdRPs in 4 families under *Picornavirales* in the database. X-axis: the names of the 4 families. The blocks with different sizes and colors in the bar represent the genera under the corresponding family.

The performance of read classification at the family and genus levels under *Piconavirales*.

| Taxon | taxonomic level for classification | # of reference RdRps | # of testing samples | Precision | Recall | F-score | # of classes |
|---|---|---|---|---|---|---|---|
| Picornavirales | family | 168 | 1273 | 0.971 | 0.970 | 0.970 | 4 |
| Caliciviridae | genus | 29 | 345 | 0.884 | 0.849 | 0.844 | 3 |
| Dicistroviridae | genus | 10 | 64 | 0.894 | 0.867 | 0.863 | 2 |
| Picornaviridae | genus | 17 | 116 | 0.994 | 0.994 | 0.994 | 2 |
| Secoviridae | genus | 37 | 322 | 0.926 | 0.898 | 0.896 | 6 |

We can see that the F-score at the family level is 0.97, and the average F-score at the genus level is 0.899. And the F-scores at the family and genus levels are smaller than that at the order level, which is 0.983 for *Piconavirales*. At the genus level, the F-score decreases because there are not enough references. For example, the family *Dicistroviridae* has 2 genera, containing only 6 and 4 RdRps respectively as references. Besides the insufficient training samples at lower taxonomic levels, frequent changes at lower taxonomic levels can also make classification performance evaluation difficult. Comparing the taxonomy provided by ICTV in 2019 with that in 2020, the number of taxa changed from 1421 to 2224 at the genus level, but only from 55 to 59 at the order level. Thus RdRpBin is designed and recommended for order-level taxonomic classification. With more stable and sufficient training samples at lower ranks, RdRpBin can be conveniently applied to lower ranks.

We have added the description and experimental results in Supplementary "Experiment on the lower taxonomic levels" .

***Comment 3***. *Page 2, line 36: remove "at read level". Abstract, line 29: should be "share low sequence similarity".*

**Response**: Thanks. We corrected them in the revision.
